# Further Evidence That Defects in Main Thyroid Dysgenesis-Related Genes Are an Uncommon Etiology for Primary Congenital Hypothyroidism in Mexican Patients: Report of Rare Variants in *FOXE1*, *NKX2-5* and *TSHR*

**DOI:** 10.3390/children8060457

**Published:** 2021-05-30

**Authors:** Miguel Angel Alcántara-Ortigoza, Iraís Sánchez-Verdiguel, Liliana Fernández-Hernández, Sergio Enríquez-Flores, Aidy González-Núñez, Nancy Leticia Hernández-Martínez, Carmen Sánchez, Ariadna González-del Angel

**Affiliations:** 1 Laboratorio de Biología Molecular, Subdirección de Investigación Médica, Instituto Nacional de Pediatría, Secretaría de Salud, Ciudad de Mexico CP 04530, Mexico; malcantaraortigoza@gmail.com (M.A.A.-O.); dralilianafernandez@gmail.com (L.F.-H.); yazzama@gmail.com (N.L.H.-M.); 2 Consulta Externa, Instituto Nacional de Pediatría, Secretaría de Salud, Programa de Maestría y Doctorado en Ciencias Médicas, Odontológicas y de la Salud, UNAM, Ciudad de Mexico CP 04530, Mexico; drairais@yahoo.com; 3 Grupo de Investigación en Biomoléculas y Salud Infantil, Laboratorio de Errores Innatos del Metabolismo y Tamiz, Instituto Nacional de Pediatría, Ciudad de Mexico CP 04530, Mexico; sergioenriquez5@yahoo.com.mx; 4 Hospital Regional Materno Infantil de Alta Especialidad de Nuevo León, Guadalupe CP 67140, Mexico; aidygn@gmail.com; 5 Laboratorio de Seguimiento del Neurodesarrollo, Instituto Nacional de Pediatría, Ciudad de Mexico CP 04530, Mexico; msanchez@correo.xoc.uam.mx

**Keywords:** congenital hypothyroidism, *FOXE1*, Mexican population, multiplex ligation-dependent probe amplification (MLPA), *NKX2-1*, *NKX2-5*, *PAX8*, polyalanine tract, protein modeling, thyroid dysgenesis, TSH receptor

## Abstract

Mexico shows a high birth prevalence of congenital hypothyroidism (CH) due to thyroid dysgenesis (TD). *PAX8* defects underlie only 1% of these cases and *NKX2-1* does not seem to be involved. Here, we analyzed other TD-related genes in 128 non-related Mexican patients (females 77.3%; 6 months to 16.6 years) with non-syndromic CH-TD diagnosis established by clinical evaluation, thyroid hormone serum profiling, and scintigraphy (74%) or ultrasonography (26%). We performed Sanger sequencing of *FOXE1*, *NKX2-5*, and *TSHR* and evaluated copy number variations (CNVs) in *TSHR*, *FOXE1*, *PAX8*, and *NKX2-1* by multiplex ligation-dependent probe amplification. Odds ratios for TD risk were explored for *FOXE1* polyalanine stretches [polyAla-rs71369530] in cases and controls (N = 116). Five rare missense changes cataloged as benign (*NKX2-5*:p.(Ala119Ser)-rs137852684), of unknown significance (*FOXE1*:p.(Ala335Gly)-rs543372757; *TSHR*:p.(Asp118Asn)-rs1414102266), and likely pathogenic (*FOXE1*:p.(Gly124Arg)-rs774035532; *TSHR*:p.(Trp422Arg)-rs746029360) accounted for 1.5% (N = 2/128) of clinically relevant genotypes (supported in part by protein modeling) in CH-TD. No CNVs were identified, nor did polyAla > 14 alanines in *FOXE1* significantly protect against TD. The present and previously published data collectively show that small clinically relevant germline variants in *PAX8*, *FOXE1,* and *TSHR* are found in only a very small proportion (2.5%) of isolated CH-TD Mexican patients.

## 1. Introduction

Congenital hypothyroidism (CH) is considered the main preventable cause of intellectual disability at birth [1]. The incidence in non-Hispanic white populations is 1 in 3533–4166 live newborns [1,2]. Overall, it is estimated that thyroid dysgenesis (TD, including athyrosis, ectopy, and hypoplasia) accounts for 71–87.3% of CH cases [3,4,5]. Great variability has been noted among different ethnicities, as exemplified by the predominance of autosomal recessive dyshormonogenetic forms in Chinese (~70%) [6] and Russian (~85%) [7] CH newborns. In the French population, ~98% of CH due to TD (CH-TD) occurs as a sporadic event [4] that has an unknown etiology in >90% of the cases [3]. However, the following observations suggest that there is a genetic component to CH-TD: (a) The well-known high birth prevalence of CH in Hispanics (1:1600–1758) [1,2], including Mexicans, in whom an evident increase (1:1373) has been recently reported by newborn screening [8]; (b) the higher female:male sex ratio (2–3:1) mainly observed in Caucasian, Hispanic, and Mexican patients [1,2,5,8]; (c) the >15-fold estimated recurrence risk for first degree relatives of a sporadic CH-TD patient [4]; (d) there are reported associations with extra-thyroidal congenital defects in Caucasian (8.2–9%) [4], Brazilian (14.9%) [9], and Mexican (23%) [10] patients; (e) subtle functional (3.9–7.8%) or developmental (1–10%) thyroid abnormalities have been identified in first-degree relatives of CH-TD patients [11,12]; and (f) the TD human phenotype has been replicated in mouse models null for a number of thyroidogenesis-related human orthologous genes (*PAX8*, *FOXE1*, *NKX2-1*, *TSHR*, etc.,) whose mono- or biallelic loss-of-function genotypes, even interacting in a polygenic model, may explain the etiology of 5–10% of CH-TD patients [3].

The introduction of massively parallel or next-generation sequencing (NGS) has expanded the mutational spectrum of dyshormonogenesis and TD candidate genes for primary permanent CH in patients who were identified through newborn screening but lacked a formal thyroid imaging evaluation [6,7,13]. This approach has proven useful in documenting the wide allelic and locus heterogeneity underlying the thyroid hormone biosynthesis defects that predominate over TD in CH patients of some backgrounds, such as Chinese [6,13] and Russian [7] ones. To date, the five extensively studied TD candidate genes, *FOXE1* (MIM*602617), *NKX2-5* (MIM*600584), *PAX8* (MIM*167415), *TSHR* (MIM+603372), and *NKX2-1* (MIM*600635), appear to convincingly explain only <10% of the CH-TD cases, even when assessed using a NGS approach [3,7]. At least six additional novel loci involved in the TD etiology have recently been described, but some are responsible for syndromic TD forms, and together the known genes still explain only a minority of patients [3]. In addition, assessing of pathogenic copy number variations (CNVs), as well as further explorations in distinct populations to French ones regarding to association of TD phenotype with *FOXE1* polyalanine stretch (polyAla) genotypes [14], has not been evaluated by the employed bioinformatics pipelines in these recent NGS studies [6,7,13]. Evaluation of CNVs by multiplex ligation-dependent probe amplification (MLPA) identified whole-exon heterozygous deletions in *PAX8*, *TSHR,* and *FOXE1* as being relevant pathogenic changes in 11% of CH-TD Polish patients [15], but this has not been replicated in other populations [7,16].

Mexico shows one of the highest and increasing worldwide CH birth prevalences [2,8], with the disease mostly attributable to TD (~93%) [17]. However, the etiology of the disease in this population remain largely elusive [8,17]. Two Sanger sequencing-based reports found that heterozygous loss-of-function *PAX8* genotypes account for the etiology of ~1% of Mexican CH-TD patients [18], and that clearly deleterious small nucleotide changes in *NKX2-1* did not appear to be a factor in this population [19]. To gain a more comprehensive picture of the main TD-related genes in a Mexican population, we herein studied 128 unrelated Mexican patients at pediatric age, all bearing a confirmed clinical, biochemical, and thyroid imaging diagnosis of non-syndromic CH-TD. In them, Sanger sequencing was applied to characterize small nucleotide changes in *NKX2-5*, *TSHR,* and *FOXE1* (including genotyping of the polyAla stretch proposed as a TD susceptibility polymorphic variant [14]) and MLPA to assess CNVs in *TSHR*, *FOXE1*, *PAX8,* and *NKX2-1*. As has been described for most populations [3], our results indicate that germline defects in the studied TD-related genes are a very uncommon or even a debatable genetic cause for the majority of Mexican CH-TD patients, and suggest that a single-gene model does not explain the high birth prevalence of this congenital disorder in Mexico.

## 2. Materials and Methods

### 2.1. Population Study

During 2013–2015, we recruited 128 non-related Mexican patients (99 females, 77.3%) at pediatric age (6 months–16.6 years of age), all with a confirmed diagnosis of non-syndromic, primary and permanent CH-TD, as evaluated by a pediatrician (I.S.-V.), neurodevelopmental specialist (C.S.), endocrinology pediatrician (A.G.-N.), and clinical geneticists (A.G.-d.A., L.F.-H.). Identification of CH relied on newborn screening in 89% (N = 114/128) of participants. Further confirmation of CH-TD was established by the presence of a thyroid hormone serum profile indicating hyperthyrotropinemia (>10 µUI/mL) and low total and free T4 (<4.5 mcg/dL and <0.8 ng/dL, respectively), as assessed in most of the patients (74%, N = 96/128) at ages 31–60 days after birth, as well as by the presence of an abnormal thyroid anatomy diagnosed in 74% (N = 95/128) of patients by scintigraphy with radionuclide uptake of sodium pertechnetate 99 m (Tc99 m) or by thyroid ultrasonography (26%, N = 33/128), which revealed 45.3% (N = 58/128) instances of thyroid ectopy, 42.2% (N = 54/128) of athyrosis, and 12.5% (N = 16/128) of thyroid hypoplasia. Parental consanguinity was not referred to in any patient. In 14 families (10.9%), different thyroid disorders (CH, thyroid neoplasms, non-specified hypothyroidism, etc.,) were documented in second- or third-degree relatives and, in three cases, the mother endorsed primary (N = 2) or unspecified hypothyroidism (N = 1). Diverse central nervous system (N = 4), ophthalmic (N = 4), cardiovascular (N = 3), genitourinary (N = 2), orthopedic (N = 6), and hematologic (N = 1, factor XII deficiency) abnormalities were documented in 15.6% of patients, but no recognized syndromic form of CH-TD was established during their clinical genetic evaluations. One familial case of neurofibromatosis type 1 (MIM#162200) was identified in a patient (HC-222) and her mother. A normal Sanger sequencing result for the *NKX2-1* gene was previously reported for 122 of the 128 patients included herein [19].

Written informed consent was obtained from parents of all included participants. This study was approved by the Research, Biosecurity and Ethics committees of the National Institute of Pediatrics, Mexico (Registries 083/2013 and 058/2019).

### 2.2. Molecular Analysis

#### 2.2.1. Sanger Sequencing of *NKX2-5*, *FOXE1*, and *TSHR*

Genomic DNA samples were obtained from peripheral blood leukocytes by the saline precipitation method (Puregene kit; Gentra Systems, Minneapolis, MN, USA). Polymerase chain reaction (PCR) amplification and direct automated Sanger sequencing were applied to the two coding exons of *NKX2-5* gene isoform 1 along the intron–exon boundaries (NM_004387.4 and NG_013340.1 RefSeqGene), the single coding exon of *FOXE1* (NM_004473.4 and NG_011979.1 RefSeqGene) containing the polyAla stretch (rs71369530), and the 10 exons with their intron–exon boundaries of the *TSHR* gene isoform 1 precursor (NM_000369.5 and NG_009206.1 RefSeqGene). PCR oligonucleotides and sequencing conditions are presented in Table S1A.

Identified variants were classified according to the scoring proposed by the American College of Medical Genetics and Genomics and the Association for Molecular Pathology (ACMG/AMP) [20,21]. Unclassified, novel, or very-low-frequency missense changes were subjected to in silico analysis using the PolyPhen (http://genetics.bwh.harvard.edu/pph2, accessed on 8 March 2021), PROVEAN (http://provean.jcvi.org/index.php, accessed on 8 March 2021), and Pmut (http://mmb.irbbarcelona.org/PMut, accessed on 8 March 2021) programs. They were also directly searched by allele-specific PCR assays (primer sequences and PCR conditions are in Table S1B) in 146 (292 alleles) healthy and unrelated Mexican individuals.

In order to explore a possible association between the polymorphic length of the polyAla tract of *FOXE1* (rs71369530) and the CH-TD trait, we additionally genotyped the length of the polyAla stretches by Sanger sequencing in DNA samples of 116 (232 *FOXE1* alleles) unrelated, healthy, and ethnically matched individuals. We used the 14-alanine allele and the 14/14 genotype as references, given that they showed the highest frequency among CH-TD patients and healthy controls. We then compared the allelic frequencies for <14 or >14 alanines or genotypes including ≤14/≤14 or ≥14/≥14 alanines between patients and healthy controls. The odds ratio (OR) with 95% confidence interval (CI) was calculated by Fischer’s exact test, with a statistical difference assumed at a significance level of 0.05.

#### 2.2.2. MLPA Analysis of *TSHR*, *FOXE1*, *PAX8*, and *NKX2-1*

To assess CNVs, each exon of these genes was evaluated by MLPA (SALSA^®^ MLPA^®^ probemix P319-B1 Thyroid; MRC Holland, Amsterdam, The Netherlands) according to the manufacturer’s instructions. The kit do not included probes to analyze CNVs in the *NKX2-5* gene. The obtained gene dosages were evaluated using the Coffalyser.Net Software (MRC Holland, Amsterdam, The Netherlands).

### 2.3. Structural Modeling of the Human Forkhead Box E1 (FOXE1) and Thyrotropin Receptor Isoform 1 Precursor (TSHR) Proteins

Through protein in silico modeling, we further assessed the potential deleterious effects of the identified missense variants cataloged as being of unknown significance (VUS) or likely pathogenic, and lacking evidence by functional assays. To construct protein models, we used the amino acid reference sequences NP_004464.2 and NP_000360.2 for human forkhead box E1 (FOXE1) and thyrotropin receptor isoform 1 precursor (TSH receptor), respectively. Only the sequence spanning amino acids 46 to 156 was considered when we created a model for FOXE1. The 3D model of this amino acid sequence was obtained using the I-TASSER server (http://zhanglab.ccmb.med.umich.edu/I-TASSER, accessed on 8 April 2021) [22], which is based in the Local MEta-Threading-Server (LOMETS) [23] and looks for structural templates similar to the query amino acid sequence deposited in the Protein Data Bank (PDB); these are then assembled as template fragments (threaded) into a complete protein model. To define the possible implications of the rare missense variants in *FOXE1*, we used the crystallographic structure of interleukin 1 enhancer binding factor in complex with DNA [24]. This structure showed high structural similarity with the FOXE1 model (RMSD for all the Cα atoms = 0.619 Å). Once the structures were aligned, the crystallographic coordinates of the interleukin 1 protein were deleted, and the FOXE1 and DNA structures were analyzed. We were unable to model the C-terminal domain of FOXE1, where p.(Ala335Gly) is located, due to the lack of crystallographic data.

For TSH receptor modeling, we used a fragment of crystallographic structure deposited in the PDB (code: 2xwt, aminoacyl residues 24 to 257) [25]. The rest of the amino acid sequence (residues 258 to 764) was then applied to construct a 3D model using the ITASSER server and following the above described protocol.

Once we obtained the best models for both proteins, we subjected them to energy minimization with the Molecular Modeling System UCSF Chimera software [26]. The new coordinates were manually inspected, evaluated for their geometric quality, and validated with MOLPROBITY (http://molprobity.biochem.duke.edu, accessed on 8 April 2021) [27]. The electrostatic potential of the TSH receptor model was determined with the PBEQ Solver server (http://www.charmm-gui.org/?doc=input/pbeqsolver&step=0, accessed on 8 April 2021) [28]. The aminoacyl sequence of the transmembrane region of the TSH receptor model was calculated with PROTTER (https://wlab.ethz.ch/protter/start, accessed on 8 April 2021) [29], and later embedded into the membrane structure generated by the VMD software [30].

Finally, in silico mutagenesis for missense variants of the *FOXE1* and *TSHR* models was performed using the molecular graphics system, PyMOL (Molecular Graphics System, v.2.2.0 Schrödinger, LLC).

## 3. Results

**Figure 1:**
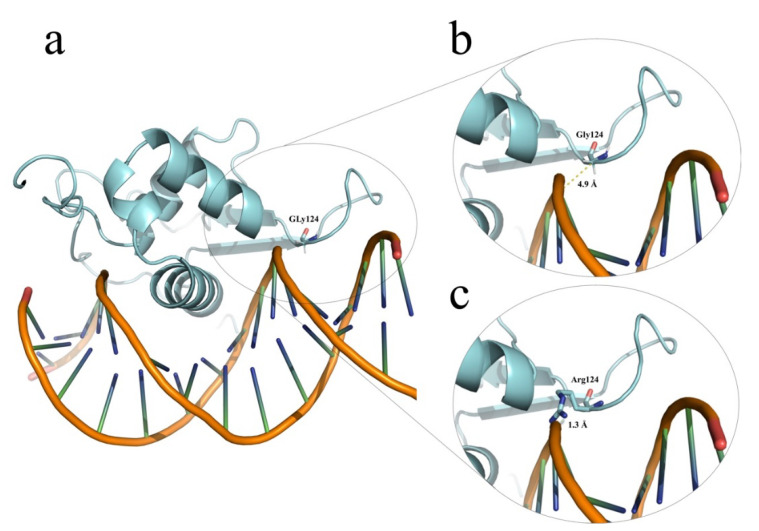
Partial FOXE1 modeling. (**a**) Schematic representations of our in silico modeling of FOXE1 in contact with DNA, as obtained with the PyMOL software. (**b**) The close-up shows a distance of 4.9 Å separating Gly124 from the minor groove of DNA. (**c**) In contrast, the model for the protein with a Gly124 to Arg124 substitution identified in HC-266 shows only 1.3 Å separating Arg124 from DNA; this leads to clash contacts, which could destabilize or alter the DNA-binding affinity of the mutant FOXE1. The 3D model was structurally aligned with the crystallographic structure of interleukin 1 (PDB code: 2C6Y).

**Figure 2:**
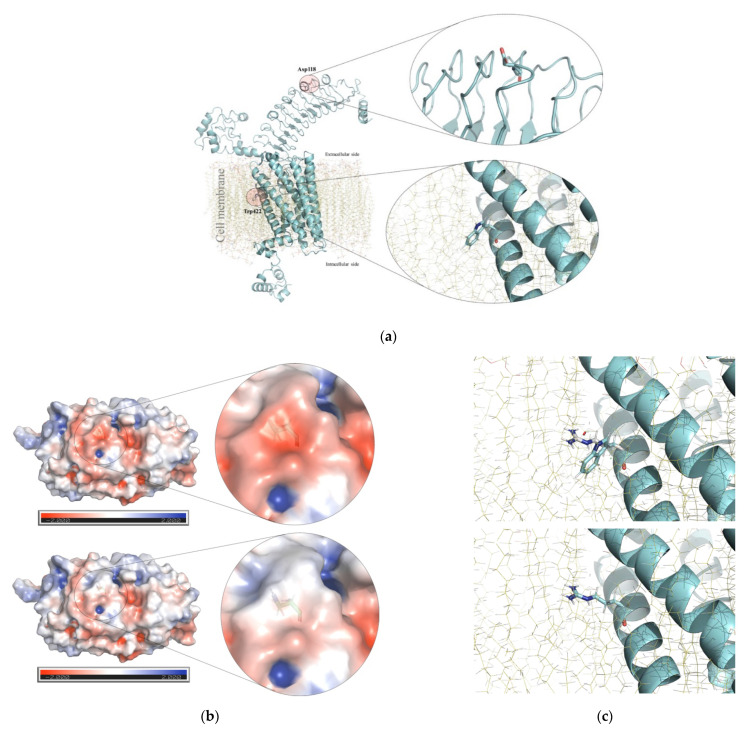
TSH receptor modeling. (**a**) Schematic representations of the complete in silico model of TSH receptor obtained with the PyMOL software, indicating the positions of Asp118 and Trp422 (including a close-up, right) involved in the substitutions identified in HC-324. (**b**) Electrostatic potential surface modeling of the convex side of the crystallographic structure (PDB code: 2XWT) of the wild-type (upper) and mutant (lower) proteins, showing that the Asp-to-Asn substitution at residue 118 induces a change from a negative (zoomed at the top) to a positive (zoomed at the bottom) electrostatic potential surface. (**c**) Close-up of position 422 in the first transmembrane domain of TSH receptor with modeling of superimposed Trp and Arg amino acids (top). The substituted amino acid residue (bottom) reveals close contact with the cell membrane.

Sanger sequencing of the analyzed genes did not reveal any patently pathogenic variant in the 128 CH-TD patients analyzed. However, five rare and single-nucleotide missense changes cataloged as benign (*NKX2-5*, N = 1), VUS (*FOXE1* and *TSHR*, N = 1 each), and likely pathogenic (*FOXE1* and *TSHR*, N = 1 each) were identified in CH-TD patients (Table 1) and were absent from healthy controls. Genotypes and clinical and thyroid phenotypes documented in these four CH-TD patients, who did not have any other extra-thyroidal congenital abnormality, are summarized in Table 1 along with other relevant data. Similarly, no CNV was identified by MLPA of the analyzed genes. The main findings of our protein modeling of p.(Gly124Arg) in *FOXE1*, p.(Asp118Asn) and p.(Trp422Arg) in *TSHR* are summarized in Figure 1 and Figure 2, respectively.

Predominance of 14 alanines in the polyAla *FOXE1* tract and homozygosity for this allele (14/14 alanines) was noted both in patients (0.86 and 75%, respectively) and healthy controls (0.87 and 78.4%, respectively), while the 16 alanine tract and 14/16 polyAla heterozygosity were the second most common allele and genotype identified among patients (0.097 and 17.9%, respectively) and controls (0.11 and 17.2%, respectively). Rare single alleles containing 10 and 17 alanines were identified in the heterozygous state in only two CH-TD patients (bearing 10/14 and 14/17 polyAla genotypes).

Fischer´s exact test did not reveal any significant statistical difference in the distributions of the <14 versus >14 alanine alleles or the ≤14/≤14 or ≥14/≥14 genotypes between patients and healthy controls. The genotypic data and statistical associations regarding the polyAla *FOXE1* polymorphic tract are presented in Table 2.

## 4. Discussion

Hispanics [1,2], including Mexicans [8], have the highest worldwide birth prevalence of CH. In contrast to the information available in the literature for Caucasian and Asian CH patients [3,6,7,13,16], relatively little is known about the participation of genetic factors in the etiology of CH-TD in Latin-American populations. To date, limited publications are available for Brazilian [31,32] and Mexican [18,19] populations. Given the predominance of TD (~93%) in the etiology of primary permanent CH in Mexicans [17], the aim of this work was to examine whether germline small-nucleotide (*NKX2-5*, *FOXE1*, and *TSHR*) and CNV-type (*FOXE1*, *PAX8*, *NKX2-1*, *PAX8*, and *TSHR*) changes in TD-related genes play an etiological role in some Mexican CH patients lacking clinical suspicion of a syndromic form and with a confirmed TD imaging-based diagnosis. Our results showed that ~1.5% of studied CH-TD patients (N = 2/128) presented relevant *FOXE1* (HC-266) and *TSHR* (HC-324) genotypes involving single-nucleotide, missense, and likely pathogenic classified variants (Table 1). We failed to identify any CNV-type change. Our results add to previous findings that 1% of CH-TD Mexican patients could be attributed to *PAX8* missense defects [18], and there was no discernible participation of small-nucleotide *NKX2-1* pathogenic changes [19]. Collectively, these data obtained in ≥100 Mexican patients suggest that germline small-nucleotide defects in the main TD-related genes, *PAX8* [18], *NKX2-1* [19], *FOXE1*, *NKX2-5*, and *TSHR* (this work), could explain ~2.5% of the isolated CH-TD cases in Mexico, without any identified etiologic role for CNVs in *PAX8*, *NKX2-1*, *FOXE1*, or *TSHR* (*NKX2-5* was not included in the employed MLPA kit). Although this explains only a small proportion of cases, it is higher than that documented in 90 Brazilian CH-TD patients, where *PAX8*, *NKX2-5*, *TSHR*, and *HES1* (MIM * 139605) did not reveal any pathogenic or VUS change [31,32], and seems quite similar to the rate obtained in Japanese CH patients (2.0%, N = 2/102), at least 50% of whom carried a confirmed TD phenotype [16]. Future genetic studies for other novel TD candidate loci (e.g., *GLIS3*, *JAG1*, *CDCA8*, *NTN1*, *TUBB1*, and/or *THRB*) [3] could be warranted in our population.

At least in the clinical setting, the European Society for Paediatric Endocrinology Consensus Guidelines on Screening, Diagnosis, and Management of CH recommends that molecular genetic study should be preceded by a careful clinical evaluation of the CH patients along with delineation of the thyroid phenotype [33]. However, this clinical aspect was not considered in recent genetic studies involving a larger number of Russian [7] and Chinese [6,13] samples. This precluded the authors from offering an updated and reliable proportion of molecular defects in TD-related genes and led to the predominant identification of dyshormonogenesis-related genotypes. In fact, we consider that the lower frequency of extra-thyroid abnormalities identified in the present study, compared to a previous report in Mexican CH patients (15.6 vs. 23%, respectively) [10], reflects that we performed clinical evaluation to exclude the known TD syndromic forms. The inclusion of such cases may have biased the accurate estimation of mutational spectrum underlying the most common isolated TD forms in both Caucasian [4,5] and Mexican [17] CH cases. Such a bias could be exemplified by the recurrent identification of brain-lung-thyroid syndrome (MIM#610978) in other studies [34,35].

CNV-type changes in TD-related genes have not been explored in Latin American CH populations [18,19,31,32]. In a small sample of Polish CH-TD patients directly subjected to MLPA, whole-exon heterozygous deletions in *PAX8*, *TSHR*, and *FOXE1* genes surprisingly comprised 11% (N = 5/45) of the underlying TD pathogenic genotypes [15]. The two monoallelic *PAX8* gene deletions identified in the Polish study agreed with the idea that a haploinsufficiency mechanism correlates with the reported thyroid ectopy and athyrosis phenotypes [3]. However, the authors found it difficult to explain the TD phenotype in the remaining three patients bearing monoallelic *TSHR* and *FOXE1* deletions [15], especially as they did not identify a second pathogenic allele (i.e., by whole-gene sequencing) in these patients. The absence of these gene rearrangements in our larger sample of CH-TD Mexican patients agree with the data obtained from Japanese (*PAX8*, *NKX2-1*, and *FOXE1*) [16] and Russian (1/243 CH patients with *PAX8* heterozygous deletion, no deletions in *TPO*, *FOXE1*, *NKX2-1*, or *TSHR*) [7] CH patients. Thus, with the exception of the Polish experience, CNVs seem to represent an uncommon pathogenic mechanism associated with TD in several CH populations.

A recent analysis of pooled literature supported the idea that heterozygous missense variants located at *PAX8*, *TSHR*, *FOXE1*, and *NKX2-5* could increase the risk for developing permanent CH (OR = 37.38, 95% CI 5.04-277.21) [36]. In our study, we identified a total of five rare missense single-nucleotide changes in *FOXE1* (N = 2, homozygous and heterozygous genotypes), *NKX2-5* (N = 1, heterozygous genotype), and *TSHR* (N = 2, compound heterozygous genotype); of them, only two were cataloged as “likely pathogenic” variants (Table 1).

Most alleles conditioning the Bamforth-Lazarus syndrome are missense changes that impair the DNA-binding activity of the FOXE1 forkhead domain (amino acid positions 53 to 141) [37]. The Gly124 position is located just inside this domain and shows high phylogenetic conservation from human to zebrafish. The non-conservative amino acid substitution identified in HC-266 changes a small hydrophobic amino acid (glycine) to a positively charged and larger residue (arginine). Our protein modeling indicated that Gly124 is in close contact with DNA, and its drastic substitution with an arginine causes clash contacts (Figure 1), leading to a possible destabilization of the FOXE1 DNA-binding site. This modeling correlates with the in silico predictions made by the PolyPhen, PROVEAN, and Pmut programs, which unanimously predicted the p.(Gly124Arg) *FOXE1* variant as deleterious with high confidence scores. To date, the p.(Gly124Arg) *FOXE1* allele (rs774035532) exhibits a very low worldwide allelic frequency (0.0016%) and has been identified exclusively in four heterozygous Latin American individuals according to gnomAD database (https://gnomad.broadinstitute.org/variant/9-100616566-G-C, accessed on 27 May 2021). Although there have been a few reported cases of Bamforth-Lazarus syndrome, a wide variation in clinical expressivity has been noted. For example, not all described patients have choanal atresia or epiglottic bifidity, and a minority shows a thyroid hypoplasia instead of athyrosis [37], as seen in our patient HC-266. Attempts to correlate this variable clinical expressivity with the residual functional activity of mutant FOXE1 protein have not been conclusive [37], and functional studies based on gel mobility-shift and transfection assays are needed to corroborate if p.(Gly124Arg) along with a 16 alanine polyAla tract represents a hypomorphic *FOXE1* allele that retains considerable residual function to prevent the full development of Bamforth-Lazarus syndrome and only leave CH-TD as the phenotypic manifestation. To the best of our knowledge, our patient HC-266 could represent the first case of a non-syndromic CH-TD diagnosed by newborn screening and bearing a likely pathogenic biallelic *FOXE1* genotype, which expands the phenotypic spectrum for *FOXE1*-related disorders. On the other hand, it is generally assumed that the heterozygous parents of Bamforth-Lazarus syndrome patients are healthy and euthyroid [37], as was documented in the parents of HC-266. Unfortunately, we lacked information on the thyroid morphology in these obligate carriers and thyroid image evaluations could not be performed to discard any asymptomatic congenital thyroid defect. In addition to the 16/16 polyAla genotype in HC-266, we noted homozygosity at the rs201181824, rs139551528, rs371516340, and rs3021526 sites (data not shown). This could suggest an identity-by-descent for the 9q22.33 region, potentially reflecting endogamy or consanguinity of the parents (although such antecedents were denied), as has been described for most Bamforth-Lazarus syndrome families [37].

The asymptomatic carrier status for proven pathogenic *FOXE1* alleles makes it difficult to propose any etiological role for heterozygous VUS in the development of CH-TD. Such heterozygosity was seen for the p.(Pro8Arg) and p.(Leu112Phe) variants recently identified in Chinese CH patients and their healthy parents [13], and the p.(Ala335Gly) [rs543372757] herein reported for HC-215 and her euthyroid mother (Table 1). The p.(Ala335Gly) is an extremely infrequent allele worldwide (0.00076%); to date, it has been identified only in the heterozygous state (2/121660 alleles) in European (Non-Finnish)-descent populations (https://gnomad.broadinstitute.org/variant/9-100617200-C-G, accessed on 27 May 2021). The Ala335 position does not show a high degree of phylogenetic conservation, and it is located at the C-terminus of FOXE1, which is devoid of critical domains or known pathogenic variants. The p.(Ala335Gly) variant involves a conservative change, as alanine and glycine are small and hydrophobic residues; however, the in silico predictions of its effects were contradictory, ranging from tolerated to probable damaging. Unfortunately, we were unable to perform in silico modeling for the C-terminal domain of FOXE1, and thus could not use this information to consider its possible deleterious effects. Thus, future functional analysis and assessment of its cosegregation with the CH-TD trait in the homozygous state in other families are needed to determine the benignity or pathogenicity of this variant.

The knockout *Nkx2-5^-/-^* mouse model develops thyroid bud hypoplasia [38], a feature that encouraged the study of its human homologue in CH-TD patients [3,38], especially those with associated congenital heart defects (CHD) [39]. However, although *NKX2-5* has been extensively studied among different CH populations, including Chinese [13], Japanese [16], Brazilian [31,32], Czech [39], Dutch [40], and Iranian [41] patients, no fully penetrant pathogenic genotype has been found to condition CH-TD [40], with or without CHD [38,39,40], nor is TD a characteristic feature of patients with CHD attributable to *NKX2-5* defects [40]. We did not herein carry out CNV evaluation for *NKX2-5*, although it is currently thought that genetic testing of *NKX2-5* is not necessarily indicated in TD [3,40]. However, some studies [38,40,41] commonly reported two likely benign missense changes: p.(Arg25Cys) [rs28936670] and the herein-identified (HC-321) p.(Ala119Ser) [rs137852684]. The latter change is located upstream from the homeobox NKX2-5 domain (amino acid positions 145 to 194); it is an uncommon allele (0.001340) among the European populations, but homozygous individuals have been identified in South Asian populations (https://gnomad.broadinstitute.org/variant/5-172660192-C-A, accessed on 27 May 2021). This, coupled with the recent functional in vitro evidence, the lack of cosegregation with CHD, the absence of TD in heterozygous individuals, and the healthy and euthyroid status observed in the heterozygous father of HC-321, collectively support a benign feature for p.(Ala119Ser) [40]. However, it still remains to be determined if these and other documented monoallelic *NKX2-5* VUS in CH patients, i.e., p.(Ser139Asn) [6], p.(Arg143Gln) [35], p.(Pro211Leu) [34], and p.(Asp226Asn) [7], could play roles as genetic susceptibility factors, even in a digenic or polygenic model [3,42].

*TSHR*-related disorders display a broad clinical spectrum due to wide allelic heterogeneity. Monoallelic or biallelic loss-of-function variants of *TSHR* condition partial or complete TSH resistance, respectively. The first is generally manifested as mild postnatal hyperthyrotropinemia (hyperTSH) associated with an apparently euthyroid state accomplished by a normal-sized and orthotopic gland; the second is generally characterized as severe non-autoimmune CH due to thyroid hypoplasia [43]. This complete TSH resistance leads to CH identified by TSH-based newborn screening [43], as occurred with HC-324: This patient was diagnosed at 1 month of age through a marked elevation of serum TSH (>150 µUI/, reference value: 0.4-4-0 µUI/mL) along with severe hypothyroidism (total T3: 10 ng/dL, ref. value: 72–170 ng/dL; free T3: 0.01 pg/mL, ref. value: 1.8–6.0 pg/mL; total T4: 0.7 mcg/dL, ref. value: 4.5–12.5 mcg/dL; free T4: 0.4 ng/dL, ref. value: 0.8–1.9 ng/mL). Thus, it seems plausible to consider the compound heterozygous p.[Asp118Asn];[Trp422Arg] as a potential loss-of-function biallelic *TSHR* genotype. These missense variants are not currently described as being responsible for TSH resistance in ClinVar (https://www.ncbi.nlm.nih.gov/clinvar, accessed on 8 March 2021), Human Gene Mutation Database (HGMD^®^, http://www.hgmd.cf.ac.uk/ac/index.php, accessed on 8 March 2021), LOVD 3.0 (https://databases.lovd.nl/shared/genes/TSHR, accessed on 8 March 2021), or TSH Receptor Mutation Database (https://tsh-receptor-mutation-database.org/list.html, accessed on 8 March 2021), and to the best of our knowledge, they have not been previously reported as being responsible for any *TSHR*-related disorder. The p.(Asp118Asn) and p.(Trp422Arg) variants display extremely low worldwide allelic frequencies (0.00039%: https://gnomad.broadinstitute.org/variant/14-81554332-G-A and 0.0015%: https://gnomad.broadinstitute.org/variant/14-81609666-T-C, respectively; accessed on 27 May 2021) and they have been identified exclusively in heterozygous Latin American individuals. The crystallographic structure (ranging from amino acids 24 to 257) deposited in PDB (code: 2XWT) and our 3D protein model (Figure 2) indicate that p.(Asp118Asn) is located in the third leucine-rich-repeat (LRR, amino acids 66 to 221) inside the large N-terminal extracellular domain of the TSH receptor, where several pathogenic missense changes have been described as altering ligand-binding capacity and/or reducing the receptor’s cell surface expression [43]. It is well-known that G protein-coupled receptors, like TSH receptor, can exist as dimers and higher-order complexes [44], and the substitution of tyrosine 116 (located at 3.9 Å from Asp118) to serine was reported to totally abrogate the formation of multimers [45]. Since Asp118 is found on the same convex surface of LRR region 1, it seems plausible that this residue could participate in the formation of TSH receptor multimers. Additionally, the p.(Asp118Asn) variant involves the substitution of a negatively charged surface amino acid for a positively charged residue and is predicted to alter the surface electrostatic potential (Figure 2), which largely determines the proper electrostatic interactions of TSH receptor with its ligand and/or blocking antibodies [25,46].

The p.(Trp422Arg) variant is a non-conservative substitution that changes a polar, hydrophobic (tryptophan) residue to a positively charged (arginine) one; it affects a phylogenetically invariable position and could disturb the hydrophobic milieu of the first transmembrane domain (TM helix 1, amino acids 416 to 442, Figure 2), where substitutions close to Trp422 have been reported to interfere with phospholipase C-activating G-protein coupled receptor signaling [43,47]. Regardless of the classification of these variants according to ACMG/AMP criteria (Table 1) and protein modeling predictions (Figure 2), further functional studies performed in vitro will be needed to determine if they impair some of the described cellular mechanisms governed by TSH receptor.

Profound thyroid hypoplasia without thyroid Tc99 m uptake is characteristic of complete TSH resistance [43], which contrasts with the athyrosis phenotype documented in HC-324 by ultrasonography at 18 months of age. Unfortunately, the lack of serum thyroglobulin in HC-324 does not allow to disclose the absence of thyroid tissue, and several factors (i.e., younger age) could interfere with the accurate delineation of a thyroid phenotype by ultrasonography, where it has been estimated that up to 18% of the cases could be misdiagnosed mainly as athyrosis [48].

To further support the pathogenicity of the *TSHR* variants identified in HC-324, *TSHR* genotyping and complete thyroid profiling should be performed in both parents to assure their obligate heterozygous status, and assessment for possible partial and non-autoimmune TSH resistance [43] should be performed. Even if we assume that the identified p.[Asp118Asn];[Trp422Arg] variants represent a clinically relevant *TSHR* genotype, these defects seem to be a very uncommon genetic etiological factor for CH-TD in our population (<1%, N = 1/128). This contrasts with the situation in various other populations, where biallelic or monoallelic *TSHR* genotypes are estimated to account for around 4–9% of CH cases [43]. However, it should be noted that the highest reported proportions have been documented in small samples of Italian (~7%, N = 1/14) [49], Macedonian-Albanian (14%, N = 2/14) [50], and multiethnic CH patients that were enriched for familial forms (2.9%, N = 1/34 families) [51] showing an in situ thyroid gland. This contrasts with our study population, as most of the included patients (87.5%) presented ectopy and athyrosis.

Finally, we explored whether the polyAla *FOXE1* tract was associated with the TD trait in our population. In a previous case-control study and transmission disequilibrium test in French Caucasians, the 16 alanine allele (either 14/16 heterozygous or 16/16 homozygous status; allele dose effect) was identified as a protective TD factor (0.39, 95% CI = 0.22–0.68, *p* = 0.0005) [14]. This contrasted with a TD risk of OR 3.02 for the 14/16 genotype observed in Sicilian primary permanent CH patients [52]. Despite these contradictory data, which suggest that the polyAla *FOXE1* tract should be studied in other populations, recent NGS-based studies in Chinese [6,13] and European [7,34] CH cohorts did not explore the association of polyAla *FOXE1* alleles and/or genotypes with the risk of TD. This may reflect: the inherent difficulties of using NGS bioinformatics approaches to achieve accurate variant annotation in repetitive sequences, such as polyAla stretches; the high GC content of the *FOXE1* gene (~73%), which could lead to a low sequencing depth and coverage (i.e., 51.7%) [13]; and/or the inclusion of CH patients regardless of their thyroid phenotype [6,7,13]. Unlike the analysis carried out by Carré A et al. [14], which considered only the 14 and 16 alanine alleles and genotypes, our association approach encompassed all identified polyAla alleles and genotypes, including a novel 10 alanine allele and a rare 17 alanine allele. While a tendency for a protective effect was noted for alleles larger than 14 alanines and their genotypes, similar to that described for French Caucasian patients [14], this effect did not achieve statistical significance (Table 2). Thus, alleles larger or shorter that 14 alanines or their genotypes did not appear to influence the risk for developing CH-TD in our population. Future association analyses performed with larger samples of patients and controls among different ethnicities may identify strong and specific associations between polyAla *FOXE1* stretches and specific thyroid phenotypes [14,52,53], sex [14], extra-thyroidal congenital defects [52], and/or familial CH-TD antecedents [53].

## 5. Conclusions

- Although the Mexican population has one of the highest worldwide CH birth prevalences, our results along with the previously published findings confirm that small nucleotide and clinically relevant germline variants in the main TD-related genes of *PAX8* [18], *NKX2-1* [19], *FOXE1*, *NKX2-5*, and *TSHR* account for a minority (2.5%) of primary and permanent CH Mexican patients due to non-syndromic TD.
- Two previously unreported and clinically relevant genotypes, homozygous *FOXE1* p.[Gly124Arg];[Gly124Arg] and compound heterozygous *TSHR* p.[Asp118Asn];[Trp422Arg], were found to account for 1.5% (N = 2/128) of the CH-TD patients, but further functional and segregation analyses are warranted.
- Unlike Polish CH patients [15], Mexican CH-TD patients did not harbor CNVs in *PAX8*, *NKX2-1*, *FOXE1*, or *TSHR*.
- Although we did not evaluate CNVs in *NKX2-5*, our data support the idea that it has negligible etiological participation in TD along with a benign character for its p.(Ala119Ser) allele [40].
- The protective effect on TD risk previously described for *FOXE1* polyAla alleles larger than 16 alanines or their genotypes in French Caucasians [14] was not significantly supported in our Mexican CH-TD population.

## Figures and Tables

**Table 1:** Genotypic and clinically relevant data identified by Sanger sequencing of *NKX2-5*, *FOXE1*, and *TSHR* genes among 128 Mexican unrelated CH-TD patients.

Patient ID (Gender)	Genotype	ACMG/AMP ^1^ Criteria and Variant Classification GnomAD Allele Frequencies ^2^	Thyroid and Clinical Phenotype	Relevant Familial History
HC-266 (female)	Homozygous NM_004473.3 (FOXE1):c.370G>C or p.(Gly124Arg) [rs774035532] at forkhead domain. Homozygous for 16 alanine polyAla tract.	PM1, PM2, PP2, PP3. Likely pathogenic (V). 0.000016	Thyroid hypoplasia (scintigraphy) without other birth defects or clinical data suggestive of Bamforth-Lazarus syndrome (MIM#241850).	No familial history of CH. Healthy apparently non-consanguineous parents with normal thyroid profiles. Heterozygous for p.(Gly124Arg) and 14/16 alanine polyAla *FOXE1* genotypes.
HC-215 (female)	Heterozygous NM_004473.3 (FOXE1):c.1004C>G or p.(Ala335Gly) [rs543372757]. Heterozygous for 14/16 alanine tract.	PM2, PP2. Variant of unknown significance. 0.0000076	Thyroid ectopy (scintigraphy) without other birth defects.	No familial history of CH. Healthy non-consanguineous parents with normal thyroid profile. Mother heterozygous for c.1004C>G or p.(Ala335Gly).
HC-321 (male)	Heterozygous NM_004387.3 (NKX2-5):c.355G>T or p.(Ala119Ser) [rs137852684].	PM2, PP2, BP6, BS2, BS4. Benign (II). 0.00097	Thyroid ectopy (scintigraphy) without other birth defect.	No familial history of CH or congenital heart disease. Healthy non-consanguineous parents with normal thyroid profile. Father heterozygous for c.355G>T or p.(Ala119Ser) without any clinical data suggestive of heart disease.
HC-324 (female)	Compound heterozygous NM_000369.2(TSHR) c.[352G>A];[1264T>C] or p.[Asp118Asn];[Trp422Arg] (rs1414102266 and rs746029360).	p.(Asp118Asn): PM2, PP2, BP4. Variant of unknown significance. 0.0000039 p.(Trp422Arg): PM1, PM2, PP2, PP3. Likely pathogenic (V). 0.000015	Thyroid agenesis (ultrasonography); serum thyroglobulin levels not available.	No familial history of CH. Healthy non-consanguineous parents not available for thyroid profile evaluation or *TSHR* genotyping.

^1^ Accordingly to the available online tool https://www.medschool.umaryland.edu/genetic_variant_interpretation_tool1.html/ (accessed on 8 March 2021) [21], which is based on the American College of Medical Genetics and Genomics and the Association for Molecular Pathology standards and guidelines for the interpretation of sequence variants [20]. ^2^ Worldwide allelic frequencies reported at gnomAD database v2.1.1 (https://gnomad.broadinstitute.org, accessed on 27 May 2021).

**Table 2:** Fisher’s exact test results for *FOXE1* polyAla alleles and genotypes between CH-TD and healthy controls.

PolyAla Allele	CH-TD Alleles (N = 256)	Allelic Frequencies	Healthy Control Alleles (N = 232)	Allelic Frequencies	OR (_95%_ CI)	*p*-Value
<14 alanines	8	0.03125	2	0.0172	3.67 (0.77–17.5)	0.110
14 alanines (reference)	222	0.8671	204	0.8793		
>14 alanines	26	0.1015	26	0.1120	0.91 (0.51–1.63)	0.883
**PolyAla genotype**	**CH-TD patients (N = 128)**	**Frequency (%)**	**Healthy controls (N = 116)**	**Frequency**	****OR** (**_95%_ CI**)**	***p*** **-Value**
≤14/≤14 alanines	7	5.5	2	1.7	3.31 (0.67–16.39)	0.174
14/14 alanines (reference)	96	75	91	78.5		
≥14/≥14 alanines	25	19.5	23	19.8	0.91 (0.54–1.94)	1.0

Abbreviations: CI: confidence interval; CH-TD: congenital hypothyroidism by thyroid dysgenesis; OR: odds ratio.

## Data Availability

Publicly available datasets were analyzed in this study. This data can be found here: ClinVar: https://www.ncbi.nlm.nih.gov/clinvar/, accessed on 8 March 2021; dbSNP: https://www.ncbi.nlm.nih.gov/snp/, accessed on 8 March 2021; Genome Aggregation Database (gnomAD) v.2.1.1: https://gnomad.broadinstitute.org/, accessed on 27 May 2021; LOVD: https://www.lovd.nl/, accessed on 15 March 2021; OMIM: https://www.omim.org/, accessed on 8 March 2021; NCBI: https://www.ncbi.nlm.nih.gov/gene, accessed on 8 March 2021; Protein Data Bank (PDB): http://www.wwpdb.org/, accessed on 8 April 2021; UNIPROT: https://www.uniprot.org/, accessed on 8 April 2021. The data presented in this study are available on reasonable request from the corresponding author. The clinical and molecular data of patients and their relatives are not publicly available due to restrictions to preserve their confidentiality, which was part of the signed informed consent of each patient. All the herein reported clinically relevant genetic variants along with the available deidentified phenotypic data were submitted to the publicly available database LOVD v.3.0 - Leiden Open Variation Database (https://www.lovd.nl/, accessed on 15 March 2021).

## Supplementary Materials

The following are available online at https://www.mdpi.com/article/10.3390/children8060457/s1, Table S1: PCR primer sequences and sequencing conditions.
