# Supplementary material for: Further Evidence That Defects in Main Thyroid Dysgenesis-Related Genes Are an Uncommon Etiology for Primary Congenital Hypothyroidism in Mexican Patients: Report of Rare Variants in FOXE1, NKX2-5 and TSHR
Source: Children (Basel). 2021 May 30;8(6):457. doi: 10.3390/children8060457 (PMC8227333; doi:10.3390/children8060457)
Supplement: Supplementary file 1 [file children-08-00457-s001.zip › children-1205467-supplementary updated.pdf]

children

MDPI

Supplementary

Table S1-A. PCR oligonucleotides and sequencing conditions for *NKX2-5*, *FOXE1*, and *TSHR*.

PCR and internal sequencing oligonucleotides

NKX2-5 *(NM_004387.4 and NG_013340.1 RefSeq-Gene)*	Sequence (5' to3')
NKX2.5-EX1-(F)-M13F	GTAAAACGACGGCCAGTGGCACCATGCAGGGAA-GCTG
NKX2.5-EX1-R	CACCAGGCATCTTACATTCTGAAC
NKX2.5-EX2-(F)	CCACGAGGATCCCTTACCATTA
NKX2.5-EX2-(R)-M13F	GTAAAACGACGGCCAGTGGTCTCCGCAGGAG-TGAATG
FOXE1 *(NM_004473.4 and NG_011979.1 RefSeqGene)*	
M13F-FOXE1-F1-F	GTAAAACGACGGCCAGTCTGAGCTCTCCGCAGAAGG
FOXE1-F1-R	CGCGGGGTAGTAGACTGGAG
M13F-FOXE1-F2-F	GTAAAACGACGGCCAGTCGCCGTCTATGCAGGCTAC
FOXE1-F2-R	GAACGTGTGAACAGCCGATG
TSHR *(NM_000369.5 and NG_009206.1 RefSeqGene)*	
TSHR-EX1F	GAGGATGGAGAAATAGCCCCGAG
TSHR-M13F-EX1R	GTAAAACGACGGCCAGTCAC-TACTTCGGGCTGTTATTGAG
TSHR-M13F-EX2F	GTAAAACGACGGCCAG-TTAAGGTGAATTATTAGAAAAGC
TSHR-EX2R	CTTGATAGAACACGTTTAGAGAA
TSHR-M13F-EX3F	GTAAAACGACGGCCAGTG-CAGAATCCATGAGGGTTGT
TSHR-EX3R	AGAAACCAGCTCCCATTGCC
TSHR-M13F-EX4F	GTAAAACGACGGCCAGTACCCTGTGGCG-TAAATGCATA
TSHR-EX4R	CCCGACCCAGGCTATACACCATT
TSHR-M13F-EX5F	GTAAAACGACGGCCAGTAGTTTGACTACAGGTT-GTCTTC
TSHR-EX5R	GCTTTACTTATCTTCAACCTACC
TSHR-M13F-EX6F	GTAAAACGACGGCCAG-TTATTGTGTCCTGTTATTTAAGTGCATA
TSHR-EX6R	GTACTCTATAGAGTATATATGATAAGG
TSHR-M13F-EX7F	GTAAAACGACGGCCAGTTGGGATACATATGTGG-GACCTG
TSHR-EX7R	TGTTGGTCACACTAACTCTGG
TSHR-M13F-EX8F	GTAAAACGACGGCCAGTTGGTCACATTTTATTCTGA-TATTTGT
TSHR-EX8R	CTCCCCTTAATGTCTCCATTTATTCC
TSHR-EX8RSeq	ATATTCTTTTGTATGTCTTACTC
TSHR- EX9F	TATCCATCCCTCCTTAGACCAGAA
TSHR- M13F-EX9R	GTAAAACGACGGCCAGTACTGTGTCCCATGGAT-TTCCTGT
TSHR-M13F-EX10F	GTAAAACGACGGCCAGTTGGCACTGACTCTTTTCTGT
TSHR-EX10R	GTGTCATGGGATTGGAATGC
TSHR-EX10FSeq	ACTGTCTTTGCAAGCGAGTT
TSHR-EX10RSeq	GTCCATGGGCAGGCAGATAC

Note: Underlined sequences indicate the attached universal M13F sequence.

Overview of PCR and Sanger sequencing conditions:

All reactions were carried out in 30 µl under the standard conditions recommended for HotStarTaq® DNA Polymerase (www.qiagen.com/HB-0452, QIAGEN GmbH, Hilden, GERMANY), with 0.1 µM of each primer. The utilized annealing temperature was 60ºC for most reactions, except for the *FOXE1* (63ºC) and exon 10 *TSHR* (66ºC) amplicons, which also used final concentrations of 1X and 0.5X, respectively, of Q-Solution 5X (QIAGEN GmbH, Hilden, GERMANY). A modified dNTP 10 mM mix containing 25% of deaza-dGTP and 75% of dGTP (ratio 1:3) was employed to achieve better sequence results for the *FOXE1* amplicons, which possess a high GC content (>70%). All PCR products were evaluated by agarose gel electrophoresis, subjected to further enzymatic purification (ExoSAP-IT® PCR Product Cleanup, Affymetrix, Inc., Santa Clara, CA, USA), and then unidirectionally sequenced with the universal M13F primer (5'-GTAAAACGACGGCCAGT-3') and Big Dye® Terminator Cycle Sequencing chemistry (Life Technologies Corp.; performed at PSOMAGEN INC. Rockville, Maryland, USA). Genotypes were determined by direct visualization of each electropherogram after its annealing with reference sequences using Chromas Pro Version 1.7.7. (Technelysium Pty Ltd.) and with a Phred score >40. Ambiguous results were resolved by performing opposite-strand sequencing with the complementary primer (reverse or forward without the M13F sequence) or internal sequencing primer (TSHR-EX8RSeq, TSHR-EX10Fseq, and TSHR-EX10RSeq). Variant annotation compliant with Human Genome Variation Society nomenclature (https://varnomen.hgvs.org/) was performed using the Alamut® Visual version 2.14 software (SOPHiA GENETICS, Lausanne, Switzerland).

Supplementary Table S1-B. Oligonucleotides and PCR conditions employed to search rare variants in healthy and ethnically matched controls by means of allele-specific PCR assays.

GENE VARIANT	PRIMER NAME, SEQUENCE, AND OPTIMAL ANNEALING TEMPERATURE
***FOXE1* NM_004473.3:**c.370G>C or p.(Gly124Arg) at forkhead domain. NP_004464.2:p.(Gly124Arg)	**Gly124Arg-FOXE1-Fwd**<br>**5´-CTGGCTACCGTGAAGGAAGA-3´**<br><br>**WT-Gly124-FOXE1-R**<br>**5´-AGCGCCCAGTAGTTGCC-3´**<br><br>**MUT-Arg124-FOXE1-R**<br>**5´-GCGCCCAGTAGTTGCG-3´**<br><br>**ANNEALING TEMPERATURE: 61º C**
***FOXE1* NM_004473.3:**c.1004C>G or p.(Ala335Gly). NP_004464.2:p.(Ala335Gly)	**WT-Ala335-FOXE1-F**<br>**5´-CGGCCAGTTCGGAGC-3´**<br><br>**MUT-Gly335-FOXE1-F**<br>**5´-CGGCCAGTTCGGAGG-3´**<br><br>**Ala335Gly-FOXE1-Rev**<br>**5´-AACGTGTGAACAGCCGATG-3´**<br><br>**ANNEALING TEMPERATURE: 64º C**
***NKX2-5* NM_004387.3:**c.355G>T or p.(Ala119Ser). NP_004378.1:p.(Ala119Ser)	**NKX2-5-Fwd**<br>**5´-AGTGCACTTGGCAGAGTGAG-3´**<br><br>**WT-Ala119-NKX2-5-R**<br>**5´-TCTCCAGCTCCACCGC-3´**<br><br>**MUT-Ser119NKX2-5-R**<br>**5´-CTTCTCCAGCTCCACCGA-3´**<br><br>**ANNEALING TEMPERATURE: 64º C**

***TSHR* NM_000369.2:**c.352G>A or p.(Asp118Asn).<br>NP_000360.2:p.(Asp118Asn) [EXON 4]	**ARMS-TSHR-EX4R-NL**<br>**5´-GCTCTTTGAGGGCATCAGGGTC-3´**<br><br>**ARMS-TSHR-EX4R-NL-B**<br>**5´-GCTCTTTGAGGGCATCAGAGTC-3´ ***<br><br>**ARMS-TSHR-EX4R-D118N-B**<br>**5´-GCTCTTTGAGGGCATCAGAGTT-3´ ***<br><br>**ANNEALING TEMPERATURE: 65º C**
***TSHR* NM_000369.2:**c.1264T>C or p.(Trp422Arg).<br>NP_000360.2:p.(Trp422Arg) [EXON 10]	**ARMS-TSHR-EX10F**<br>**5´-GGGGACAGTGAAGACATGGTGT-3´**<br><br>**ARMS-TSHR-EX10R-NL-B**<br>**5´-GCCAGCAGACTAACGAGCCA-3´ ***<br><br>**ARMS-TSHR-EX10R-W422R-B**<br>**5´-GCCAGCAGACTAACGAGCCG-3´ ***<br><br>**ANNEALING TEMPERATURE: 65º C**

* Underlined nucleotides: Introduced mismatches to increase genotyping astringency of PCR assays.

Overview of allele-specific PCR assays:

All reactions were carried out in 30 µl under the standard conditions recommended for HotStarTaq® DNA Polymerase (www.qiagen.com/HB-0452, QIAGEN GmbH, Hilden, GERMANY), with 0.1 µM of each primer and 10-30 ng genomic DNA. Reactions were carried out with 25-30 PCR cycles, and the presence of amplicons was determined by agarose gel electrophoresis. All assays included a positive DNA control for each genotype (heterozygous patient and normal homozygous healthy control).
